# Ex-vivo evaluation of clinically-set hydraulic sealers used with different canal dryness protocols and obturation techniques: a randomized clinical trial

**DOI:** 10.1007/s00784-024-06006-5

**Published:** 2024-10-27

**Authors:** Nawar Naguib Nawar, Mohamed Mohamed Elashiry, Ahmed El Banna, Shehabeldin Mohamed Saber, Edgar Schäfer

**Affiliations:** 1 Department of Endodontics, Faculty of Dentistry, The British University in Egypt (BUE), 24 Hasan Elgamal street El Sherouk City, Nasr City, Cairo 11837 Egypt; 2 Department of Endodontics, Faculty of Dentistry, Ain Shams University, Cairo, 11566 Egypt; 3 Department of Endodontics, Dental College of Georgia, Augusta University, Cairo, USA; 4 Department of Biomaterials, Faculty of Dentistry, Ain Shams University, Cairo, 11566 Egypt; 5 Centre for Innovative Dental Sciences (CIDS), Faculty of Dentistry, The British University in Egypt (BUE), El Sherouk City, 11837 Egypt; 6 Central Interdisciplinary Ambulance in the School of Dentistry, University of Münster, Waldeyerstr. 30, D-48149 Münster, Germany

**Keywords:** Bioceramic sealers, Hydration, Hydraulic sealers, Intracanal voids, Intracanal moisture, Obturation, Single cone

## Abstract

**Objectives:**

This 2-part randomized parallel triple-blind clinical trial adopts a unique model assessing clinically-set hydraulic calcium silicate-based sealers (HCSBS) after different root canal dryness protocols and obturation techniques.

**Methods:**

For the first phase of the study, 24 teeth scheduled for orthodontic extractions were allocated into four groups according to the canal dryness protocol and the obturation technique. G1 (CLC-AHP): cold lateral compaction (CLC) with AH Plus sealer, G2 (CLC-ES-SD): CLC with Endosequence (ES) after standard canal(s) dryness (SD); G3 (SC-ES-SD): matching single-cone (SC) with ES after SD; G4 (SC-ES-PD): as G3 but after partial canal(s) dryness (PD). Teeth were extracted after one month of clinical service and examined for intracanal voids by micro-CT (2D & 3D). For the 2nd phase, another 24 teeth were allocated into four groups according to the root canal dryness protocol and the HCSBS used (ES or CeraSeal (CeS)). Teeth were extracted after one month and sectioned vertically for energy dispersive X-ray (EDX)/scanning electron microscope (SEM) examination. One-way ANOVA with Games-Howell post-hoc test and Chi-square test with multiple z-tests were used for statistical analysis.

**Results:**

SC-PD showed the highest percentage of voids (*p* < 0.05). MicroCT scans as well as EDX/SEM examination showed that PD resulted in significantly larger interfacial gaps (*p* < 0.001) with more hydration products at the sealer/dentin interface than SD.

**Conclusions:**

Both tested dryness protocols allowed the hydration of HCSBS and the formation of hydration products, thus standard dryness is recommended to reduce the incidence of intracanal voids.

**Clinical relevance:**

When using the single-cone obturation technique, intentional root canal moisture negatively affects the performance of HCSBS.

**Protocol Registration:**

http://www.clinicaltrials.gov, ID: NCT05808062.

## Introduction

For decades it was widely accepted that the choice of filling materials and techniques minimally impacts the final treatment outcome, and thus obturation was overshadowed by the importance of biomechanical cleaning and shaping [1, 2]. However, the last decade witnessed the introduction of clinical evidence that demonstrated how the stability of obturation components can influence the long-term success of root canal treatment, especially when taking the problems with synthetic polymer-based obturation materials (e.g., Resilon) into consideration [2–5]. This emphasizes the importance of understanding how filling materials interact in the dynamic environment of the root canal where they serve.

Since the introduction of mineral trioxide aggregate (MTA), several hydraulic calcium silicate-based sealers (HCSBS) were introduced into different modalities of endodontic treatment [6, 7]. The popularity of HCSBS is growing, and the claimed clinical superiority of warm vertical obturation techniques [2, 8] is now shifting towards obturation techniques employing HCSBS in conjunction with a single matched gutta-percha cone [9, 10]. This growing reputation is attributed to the marketing of a plethora of desirable characteristics of HCSBS including bioactivity [11], cytocompatibility [12], calcium ion release [13], physical and chemical stability [14], and an anti-bacterial potential [15], most of which are attributed to the by-products of their setting reaction [16]. This positive perception of hydraulic sealers was also consolidated by the fact that clinically they are performing well in different endodontic applications [7].

HCSBSs set via a hydration reaction in the presence of environmental fluids [7] producing different calcium minerals that contribute largely to their cytocompatibility and bioactive potential [13, 17–20]. Despite its importance, the hydration reaction of HCSBS with its subsequent products were only examined in laboratory models [21–25]. Such models lack the dynamism of the dental structures in clinical settings in terms of inherent substrate wetness and fluctuant functional and thermal loads. Also, there is no consensus on the best hydration environment required for the optimal setting of HCSBS [26, 27]. After cleaning and shaping procedures, the intra-radicular moisture is not standardized, and it could vary widely according to the root anatomy, number and size of exposed dentinal tubules and root canal dryness protocols [28]. Different levels of residual moisture in the root canal have been shown to alter the sealing properties and adhesion of root canal sealers [27, 29, 30]. Moreover, continuously emerging literature highlights how HCSBS have been marketed with scarce knowledge on their clinical performance [2] and urgently calls for clinical trials to accumulate sufficient evidence and establish standardization in their use [7].

Therefore, this study sought to evaluate the effect of partial or standard canal dryness as well as the obturation technique on the root canal filling quality of HCSBS that was allowed to set in the oral environment. The null hypothesis of the first phase was that there is no difference between canal dryness protocols or the obturation technique on the incidence of intracanal voids. This study also evaluates the effect of pre-obturation dryness protocols on the hydration reaction of two HCSBS. The null hypothesis was that there would be no effect of the dryness protocol on the intracanal voids and hydration products of two HCSBS.

## Subjects, materials and methods

### Ethical considerations

Ethical approval of the study was provided by the institution review board at the Faculty of Dentistry, The British University in Egypt, Cairo, Egypt (approval number: 21–038). The study protocol was registered in (http://www.clinicaltrials.gov) database, ID: NCT05808062). Subjects were treated in full compliance with the Helsinki Declaration (2008).

### Study design and sample size calculation

Both parts of the study were designed as parallel, triple-blinded, randomized trials, with an equal allocation ratio. A power analysis was designed based on a previous study [31] to have adequate power to apply a statistical test of the null hypothesis that there is no difference in the interfacial gaps between the tested groups. By adopting an alpha (α) level of (0.05), a beta (β) of (0.95); the minimum required sample size (n) was found to be 139 sections per group. Keeping in mind the evaluation of a minimum of 25 cross-sections per tooth [32], a minimum of 6 teeth per group was determined to obtain a minimum of 150 slices per group. Capitalizing on the volunteers enrolled in this part of the study, 6 teeth were allocated per group and the 24 teeth were used in the 3D analysis. Sample size calculations were performed using G*Power version 3.1.9.7 (Heinrich-Heine-University, Düsseldorf, Germany).

### Eligibility criteria

Inclusion criteria were healthy (ASA class 1), male and female volunteer dental graduates undergoing orthodontic treatment, and whose orthodontic treatment plan required extraction of one or more maxillary premolar. Root canals curvatures of the included teeth were ≤ 20° according to Schäfer et al. [33] and the periapical index score was 1 according to Orstavik et al. [34].

A “volunteering call” was announced beside the orthodontic clinic and the endodontics internship clinic, Faculty of Dentistry, The British University in Egypt. The call clearly stated that volunteers of dental graduates/interns are needed for a randomized trial, and that they would receive nothing in return, and that they had the right to withdraw from participation at any time. This category of candidates was chosen to ensure that they were aware of the elective nature of the procedure and its associated possibilities of complications. However, volunteers were still informed about the risks and possible complications, and signed a written informed consent. The included subjects were informed about their right to withdraw from participation at any time and that in case of any unforeseen complication during treatment, the tooth would be extracted immediately to revert to the original treatment plan of the volunteer.

Exclusion criteria were the presence of any systemic disease (ASA 2–6), pregnancy, poor oral hygiene or periodontal disease (probing depth > 3 mm) related to the treated tooth. Also, dental students of any level were not eligible to avoid including vulnerability factors.

### Groups, randomization, and blinding

Both the participants and the assessors were blinded, while the endodontist could not be blinded as to the nature of the materials and techniques used. Patients were randomized using computer-generated randomization (www.randome.org). The sequentially generated numbers were placed in opaque envelopes until it was time for the intervention. At that point, each participant was asked to choose an envelope, which would assign them to a group. The participants, the assessors and the statistician were all kept blind to the group assignments. However, the operator could not be blinded due to the specific instruments involved.

**Fig. 1:**
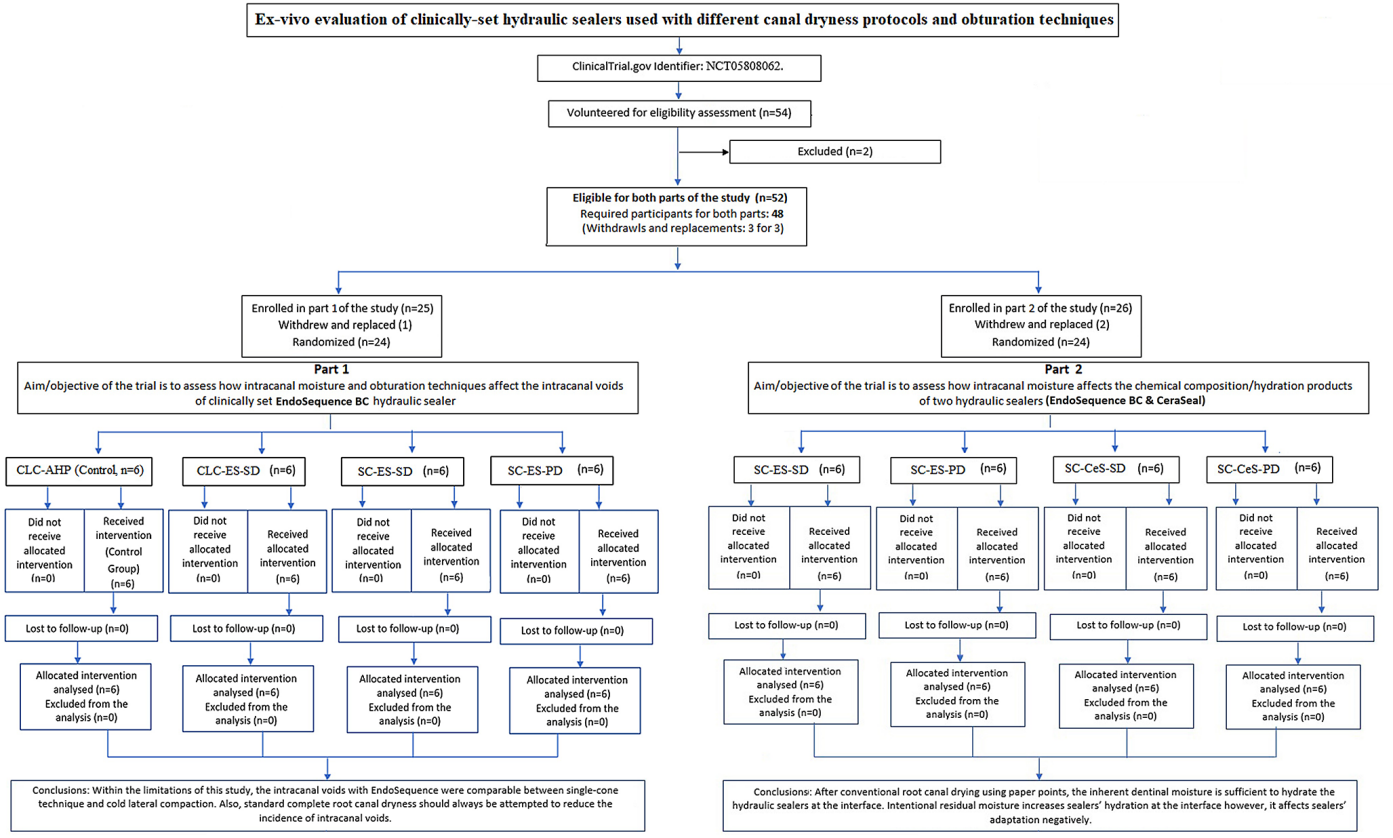
Flow chart of both phases of the study

Subjects enrolled in the 1st phase were divided into four groups; a control group and three experimental groups according to the root canal dryness protocol before obturation as well as the obturation technique (Fig. 1): G1(CLC-AHP): obturated using cold lateral compaction (CLC) and AH Plus sealer (Sirona Dentsply, Charlotte, NC, USA), G2 (CLC-ES-SD) obturated using cold lateral compaction and the hydraulic sealer EndoSequence BC Sealer (Brasseler, Savannah, GA, USA) after standard dryness (SD) using paper points attempting standard total canal(s) dryness (SD); G3 (SC-ES-SD) obturated with single-cone technique (SC) with a matching gutta-percha-cone and EndoSequence BC sealer after standard dryness; G4 (SC-ES-PD) obturated as G3 but after partial canal(s) dryness (PD). Partial dryness was not attempted with lateral compaction because it does not conform to the technique’s standards, i.e. as an obturation technique, cold lateral compaction aims at maximizing gutta-percha’s adaptation to dentin walls, with the minimal amount of sealer needed to “seal” morphological irregularities and voids between gutta-percha cones [35–38], thus the presence of any intentional residual moisture contravenes the fundamental principles of the technique.

Subjects enrolled in the 2nd phase of the study were obturated using the single-cone technique, and were divided into four groups according to the root canal dryness before obturation and the HCSBS used (Fig. 1): SC-ES-SD - obturated with EndoSequence BC Sealer after standard dryness with paper points; SC-CeS-SD - obturated with CeraSeal (Meta Biomed, Cheongju-si, South Korea) after standard dryness with paper points; SC-ES-PD - partially dried root canals obturated with Endosequence BC, and SC-CeS-PD - partially dried root canals obturated with CeraSeal.

#### Clinical endodontic procedures

A single endodontist (NNN) with more than ten years of clinical experience recorded the demographic data and completed all the clinical procedures. After anesthetizing the tooth, rubber-dam application, access cavity preparation, canal patency was confirmed and working length (WL) was determined. All root canals were shaped using the ProTaper Gold system (Sirona Dentsply) up to size F3 as per the manufacturer’s instructions. Every 3 pecking motions, the canal was irrigated with 5 ml of 2.5% NaOCl, and patency was checked. This sequence was repeated until the WL was reached. Finally, each canal was flushed with 5 mL 17% EDTA and 5 mL 2.5% NaOCl for 1 min. The total volume of NaOCl solution used throughout cleaning and shaping was standardized to be 20 ml/canal.

#### Root canal drying protocols

After a final flush with 10 ml/canal of saline solution, root canals of teeth assigned to the standard dryness groups were dried by inserting paper points of matching size to the WL till a paper point came out completely dry. In all samples, the 3rd paper point was the final paper point. In groups assigned to partial dryness, canals were dried with only one single matching-size paper point that was inserted to the WL for 5 s [29].

#### Root canal filling and coronal restoration

Root canals were obturated according to the randomization process, and the sealers were applied according to the recommendations of the manufacturers [39, 40].

For teeth obturated using lateral compaction, the technique was performed according to the literature [35–38]. The master cone was fitted to confirm the presence of a tug-back at the working length. The sealer was then introduced into the canal using a finger spreader of an equivalent size to the master cone, which was rotated counterclockwise. The master cone was then coated with the sealer and inserted to full working length. The finger spreader used in lateral compaction was chosen to reach 1 mm short of the working length [35–37]. During the obturation process, the spreader was positioned between the gutta-percha and the canal wall, and advanced apically while applying light to medium apical pressure. Upon achieving maximum penetration with the CLC technique, pressure was maintained on the spreader for a few seconds to allow the gutta-percha to deform before removing the spreader, and accessory cones were coated with sealer and placed as deeply as possible into the created space. This procedure was repeated until the spreader did not get into the radicular space, then a heated instrument was used to sear of the excess.

As for the obturation using EndoSequence BC [39], the intracanal tip was firstly attached to the syringe, then tip of the syringe was inserted into the canal, ensuring it did not extend deeper than the coronal one-third. The recommended amount of EndoSequence BC Sealer (2 calibration markings) was then gently dispensed into the root canal before a spreader was used to lightly coat the canal walls with the existing sealer. The master gutta-percha cone was then coated with a thin layer of sealer and slowly inserted into the canal, allowing it to carry the sealer to the apex. As for CeraSeal used in the second phase of the study [40]; the disposable tip was mounted on the sealer’s syringe before it was inserted into the root canal to fill the root canal without creating bubbles, before the master gutta-percha cone was inserted to the full working length gently. Given that the manufacturer did not specify the level of tip insertion other than mentioning that the root canal should be filled [40], the tip was taken to 2 mm short of the working length to allow for the sealer’s dispensation without risking periapical extrusion. Regardless of the sealer type the excess coronal gutta-percha was removed using a heated plugger and the excess sealer was removed with a cotton swab [39, 40]. All treatments were completed in the same visit before the access cavity of the tooth was permanently restored with bonded composite resin restorations.

#### Extraction

The teeth were atraumatically extracted 30 days after the root canal obturation by an oral surgeon according to the patient’s original orthodontic treatment plan. Immediately after extraction, the tooth was impermeabilized using two layers of nail varnish to seal the tooth surfaces and avoid any humidity leakage in or out [41–43] till the time of testing/scanning when the varnish would be gently scrapped. Specimens’ sterilization was done using Gamma rays because this method results in minimal heat rise if any [44, 45]. Despite precautions taken by coating the tooth and storing it in a sealed dry container, the fact that extraction was done after the obturation with a whole month allowed for logistic scheduling of different procedures, so each tooth was not stored for more than 24 h.

### First Phase

#### Teeth imaging and reconstruction

The 24 teeth were scanned using high-resolution desktop micro-CT scanner SkyScan (SkyScan 1172; SkyScan, Bruker, Belgium), then placed in a specific mold that holds the teeth in a reproducible position during the whole scanning time. The microfocus X-ray tube was set at 100 kV of acceleration voltage, and 100 µA of beam current. Scanning was performed at 13.45 μm resolution, and a 0.5 mm aluminum filter was placed for X-ray beam filtration to remove unwanted low-energy beams. The rotation step angle was 0.60°, with approximately 1350 ms of exposure time. The images were reconstructed in the same manner employed in several previous studies [46–48] using NRecon software (SkyScan 1172), with a reconstruction duration per slice set at 0.3016 s and the beam hardening correction was set at 80%.

#### Outcome assessment

Two methods were employed to assess the incidence and percentage of voids; the first method was examination of micro-CT axial cross-sections showing the occurrence of interfacial gaps directly observed and recorded by two independent examiners [31, 32], and the second method was the automatic calculation of the volume of voids at the dentin/filling interface [9, 10, 32, 49].

##### Voids assessment by direct observation of 2D cross-sections

Two experienced endodontists independently evaluated the images according to the presence or absence of gaps at the dentin/sealer interface as a binary outcome (Yes/No). Before independent assessment, the starting section and the end section were agreed upon among the blinded assessors for every tooth: The start section was determined to be the slice 0.5 mm coronal from the apical foramen while the end section was chosen as the first slice showing enamel (beginning of cementoenamel junction). The total number of sections between both slices was divided into equal intervals to obtain 25 slices or more per tooth. When the gutta-percha interface was not observable the following slices were displayed till both examiners agreed on the next section to be included. The examiners’ calibration was done the same way as described in a previous study [50]. Intra-examiner reliability was then determined using Cohen’s kappa test (Excellent, with a Cohen’s kappa value of 1), and the interclass correlation coefficient (ICC) scores were determined (ranged from 0.92 to 0.96, with a 95% confidence interval). Disagreement between the observers occurred in 24 Sect. (2.94% of the total samples) and was resolved through discussion with a third observer (an endodontist with more than 20 years of experience).

##### Percentage of voids by volume

After the 3-dimensional image reconstruction was performed, the threshold was confirmed visually and agreed upon among the blinded assessors before the automatic “grow region” feature was used. The volume of voids was calculated by subtracting the filling volume from the total volume of the root canal bounded by radicular dentin and the composite restoration [10, 49]. The percentage of voids was calculated as follows:

Voids Vol % = Total volume − Filling volume × 100/Total volume.

### Second phase

#### Teeth sectioning

A slow-speed precision diamond saw with coolant (ISOMET 4000, Bühler, Leinfelden-Echterdingen, Germany) was used for the sectioning procedures using its 0.4 mm thickness IsoMet 15 HC blade (11-4245). The roots were split vertically in a buccolingual direction producing 1 mm vertical teeth slices showing the obturation-tooth interface for other examinations. All procedures including samples preparation, testing, and calculations were done by a blinded examiner.

#### Outcome assessment

##### Scanning electron microscope/ energy dispersive X-ray (SEM/EDX) examination

Vertical root slices were gold sputtered (15 mA, 4 min) and examined using SEM and EDX at the bulk of the sealer and the sealer-tooth interface using different magnifications (1000, 3000 and 6000 X). Elemental composition analysis using EDX was performed to detect the hydration reaction products.

### Statistical analysis

Numerical data of the 3D volume of intracanal voids were represented as means, standard deviations, minimum and maximum values. Shapiro-Wilk’s test was used to test for normality. Categorical data of the interfacial gaps prevalence were presented as frequency and percentage values and were analyzed using Chi-square test followed by pairwise comparisons utilizing multiple z-tests with p-value adjustment using false discovery rate method. Homogeneity of variances was tested using Levene’s test. Data showed parametric distribution, but the variance homogeneity assumption was violated. Data were analyzed using Welch one-way ANOVA followed by Games-Howell post hoc test. The significance level was set at *p* < 0.05 within all tests. Statistical analysis was performed with R statistical analysis software version 4.3.0 (R Core Team, Vienna, Austria) for Windows.

## Results

There were no differences in gender or age among the participants in the different groups of either phase of the study as shown by Fisher’s exact test and One-way ANOVA, respectively (Table 1, *p* < 0.05).

**Table 1:** Demographic data of the participants, phase 1: CLC-AHP: cold lateral compaction with AH Plus sealer (control group), CLC-ES-SD: Cold lateral compaction and standard dryness before obturation, SC-ES-SD: single-cone and standard dryness before obturation, SC-ES-PD: single-cone and partial dryness before obturation. Phase 2: SC-ES-SD: obturated with EndoSequence BC Sealer (Brasseler, Savannah, GA, USA) after standard dryness with paper points; SC-CeS-SD: obturated with CeraSeal after standard dryness with paper points; SC-ES-PD: partially dried root canals obturated with endosequence BC, and SC-CeS-PD: partially dried root canals obturated with CeraSeal

Phase	Parameter			CLC-AHP	CLC-ES-SD	SC-ES-SD	SC-ES-PD	Test statistic	*p*-value
*1*	*Gender*	***Male***	***n***	3	4	2	4	**1.85**	**0.808** **NS**
			***%***	50.0%	66.7%	33.3%	66.7%		
		***Female***	***n***	3	2	4	2		
			***%***	50.0%	33.3%	66.7%	33.3%		
	Age (years)	***Mean ± SD***		22.33 ± 1.03	22.17 ± 0.75	22.00 ± 0.89	22.17 ± 0.75	**0.15**	**0.930** **NS**

	*Parameter*			SC-ES-SD	SC-ES-PD	SC-CeS-SD	SC-CeS-PD	*Test statistic*	*p-value*
*2*	*Gender*	***Male***	***n***	4	1	5	3	**5.87**	**0.180** **NS**
			***%***	66.7%	16.7%	83.3%	50.0%		
		***Female***	***n***	2	5	1	3		
			***%***	33.3%	83.3%	16.7%	50.0%		
	Age (years)	***Mean ± SD***		22.00 ± 0.89	22.33 ± 1.03	22.33 ± 0.52	22.17 ± 0.75	**0.23**	**0.877** **NS**

### 2D and 3D assessments of Intracanal voids

**Fig. 2:**
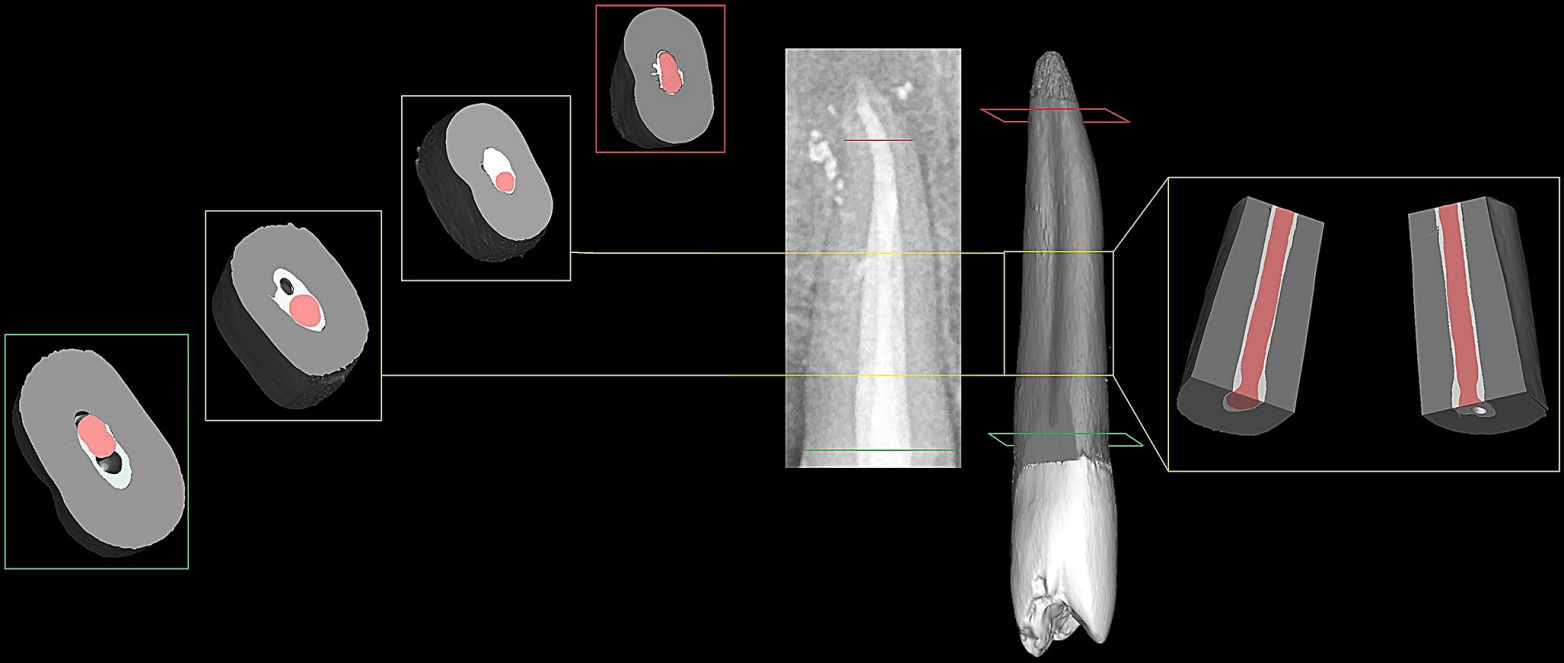
Composite figure showing the immediate postoperative radiograph of one of the included samples in the cold lateral compaction/standard dryness groups, along with its 3D constructed model, transverse cross sections, and longitudinal cross sections. Different cross sections show generally good adaptation at both interfaces, but occasional voids are seen as evident in the green and yellow cross sections

**Fig. 3:**
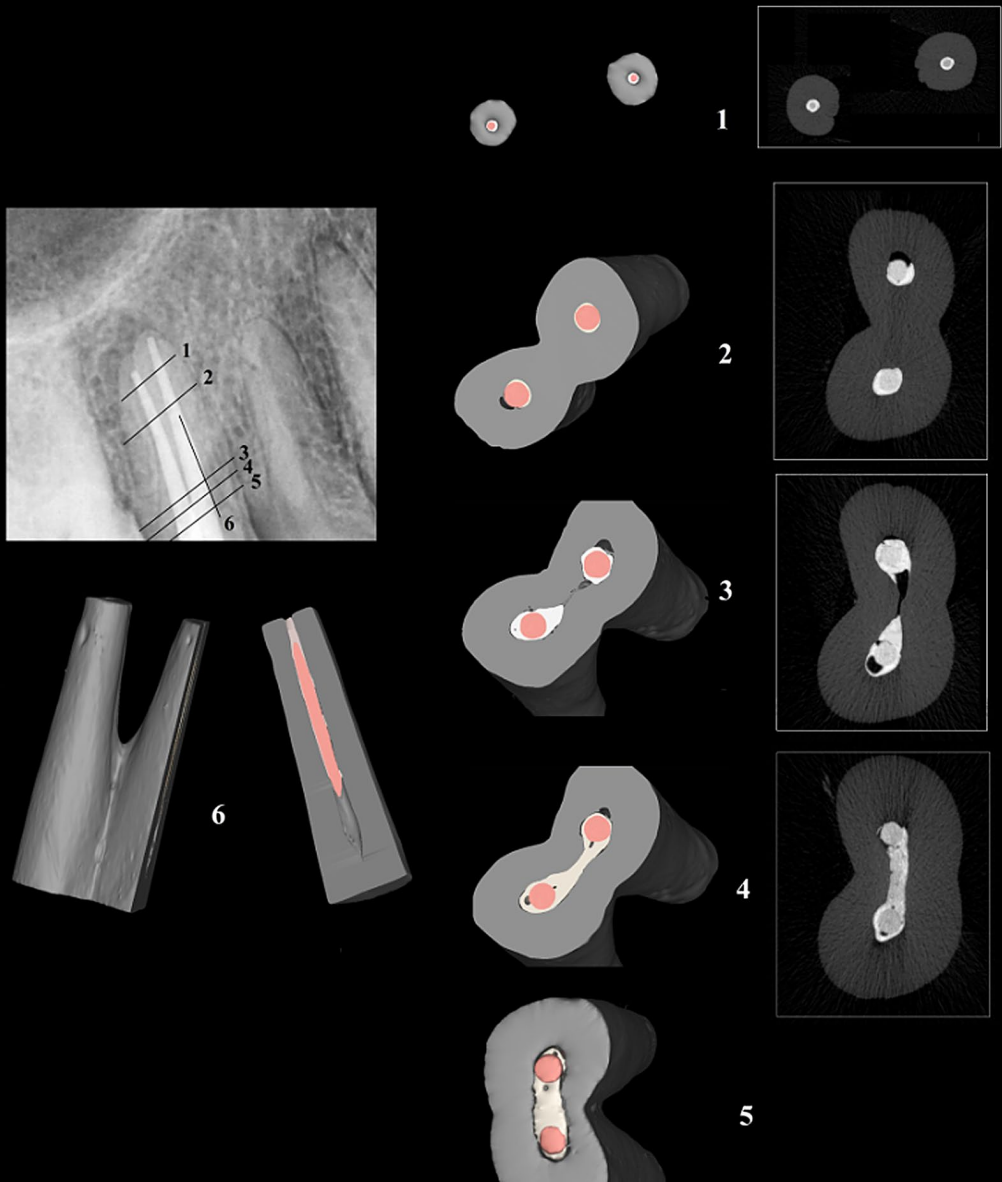
Composite figure showing the immediate postoperative radiograph of one of the included samples in the single-cone/partial dryness group, along with its 3D constructed model, transverse cross sections, and longitudinal cross sections. Except for transverse cross section-1, all slides show considerable voids at both interfaces. The greyscale of dentin only was enhanced using Adobe Photoshop (Adobe Inc, San Jose, California, United States)

**Fig. 4:**
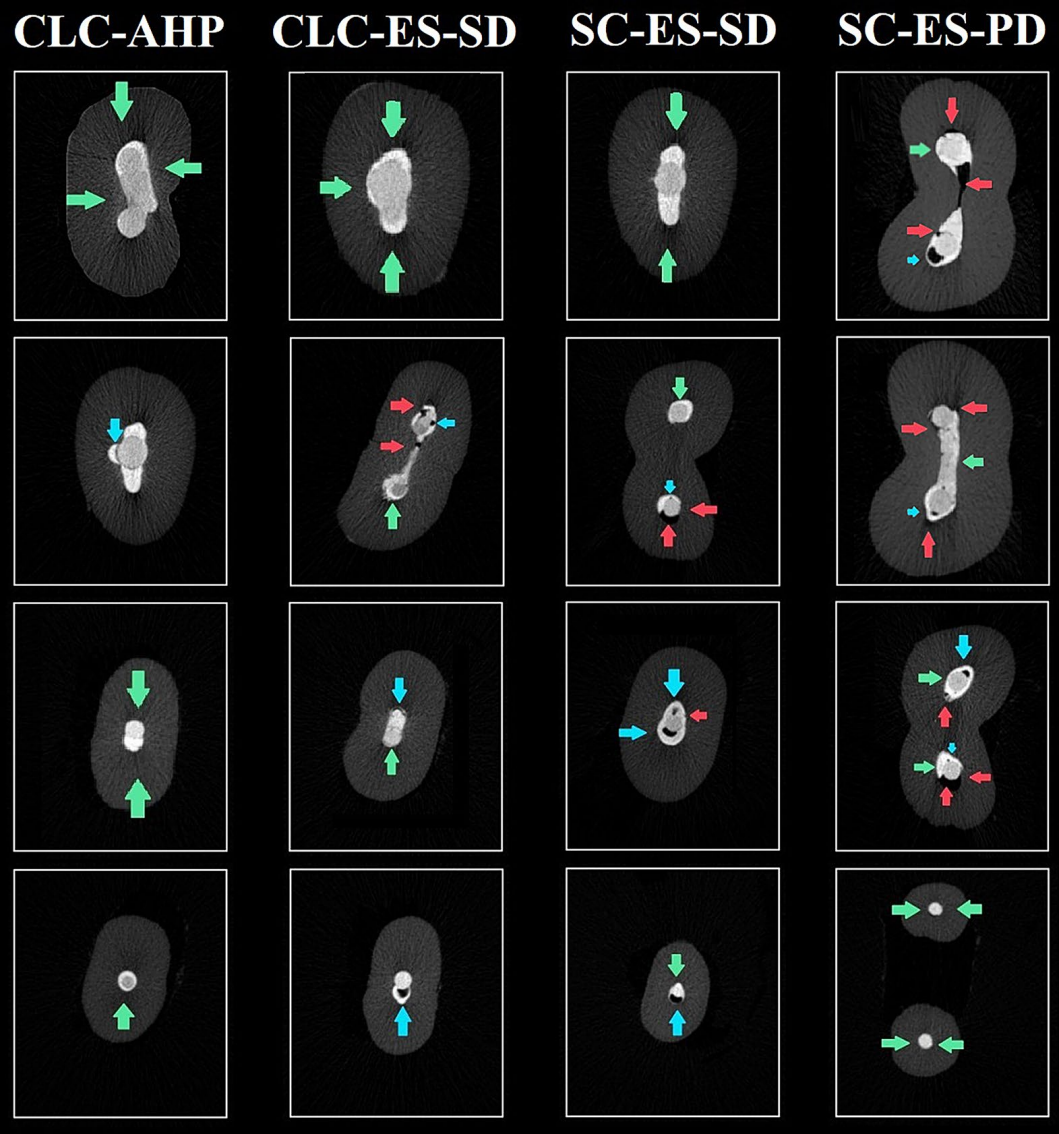
Samples of the examined axial cross-sections; green arrows show perfect adaptation on both interfaces; red arrows indicate gaps at the dentinal interface; while blue arrows indicate gaps at the sealer/gutta-percha interface. The greyscale of dentin only was enhanced using Adobe Photoshop (Adobe Inc, San Jose, California, United States)

Descriptive statistics for voids percentage are presented in Table 2 and samples are shown in Figs. 2 and 3. The intergroup comparison revealed a significant difference between the groups (*p* < 0.001) (Table 2). Post-hoc pairwise comparisons revealed that the SC-ES-PD group obtained significantly higher values than all other groups (*p* < 0.001), while the control group showed the significantly lowest values (*p* < 0.001). The difference between SC-ES-SD and CLC-ES-SD was not statistically significant (*p* = 0.995). Intergroup comparisons of interfacial gaps assessment are presented in Table 3 and samples of the examined cross sections in Fig. 4. The control group CLC-AHP showed significantly less interfacial gaps (53.1%), while there was no significant difference between CLC-ES-SD (67%) and SC-ES-SD (69.1%) denoting that the performance of single cone obturation with EndoSequence sealer matched that achieved by lateral condensation (*p* > 0.05). Finally, the SC-ES-PD group showed highly significant prevalence of interfacial gaps (82%) demonstrating the negative impact of residual moisture (*p* < 0.01). Thus, the statistical analysis of this assessment corroborated the results extracted from the 3D volumetric assessment.

**Table 2:** Descriptive statistics and intergroup comparisons for voids percentage. CLC-AHP: cold lateral compaction with AH Plus sealer (control group), CLC-ES-SD: Cold lateral compaction and standard dryness before obturation, SC-ES-SD: single-cone and standard dryness before obturation, SC-ES-PD: single-cone and partial dryness before obturation

Group	Mean		95% CI			SD		Minimum		Maximum	
			Lower		Upper						
Control	0.95		0.86		1.03	0.11		0.82		1.13	
CLC-ES-SD	2.22		2.04		2.40	0.22		1.91		2.48	
SC-ES-SD	2.26		1.97		2.56	0.36		1.76		2.68	
SC-ES-PD	13.14		11.75		14.54	1.75		10.62		15.77	
**Voids (%) (Mean ± SD)**									**f-value**		**p-value**
**Control**		***CLC-ES-SD***		***SC-ES-SD***			***SC-ES-PD***				
0.95 ± 0.11^A^		2.22 ± 0.22^B^		2.26 ± 0.36^B^			13.14 ± 1.75^C^		**140.66**		< 0.001*

95%CI = 95% confidence interval for the mean; SD = standard deviation. Means with different superscript letters within the same horizontal row are significantly different; *significant (*p* < 0.05)

**Table 3:** Intergroup comparisons of interfacial gaps presence. CLC-AHP: cold lateral compaction with AH Plus sealer (control group), CLC-ES-SD: cold lateral compaction and standard dryness before obturation, SC-ES-SD: single-cone and standard dryness before obturation, SC-ES-PD: single-cone and partial dryness before obturation

Interfacial gap	CLC-AHP		CLC-ES-SD		SC-ES-SD		SC-ES-PD		χ^2^	p-value
	*N*	*%*	*N*	*%*	*n*	*%*	*n*	*%*		
No	98^A^	46.9%	70^B^	33.0%	69^B^	30.9%	31^C^	18.0%	**36.20**	< 0.001*
Yes	111^A^	53.1%	142^B^	67.0%	154^B^	69.1%	141^C^	82.0%		

Values with different superscript letters within the same horizontal row are significantly different; *significant (*p* < 0.05)

#### SEM and EDX analysis

**Fig. 5:**
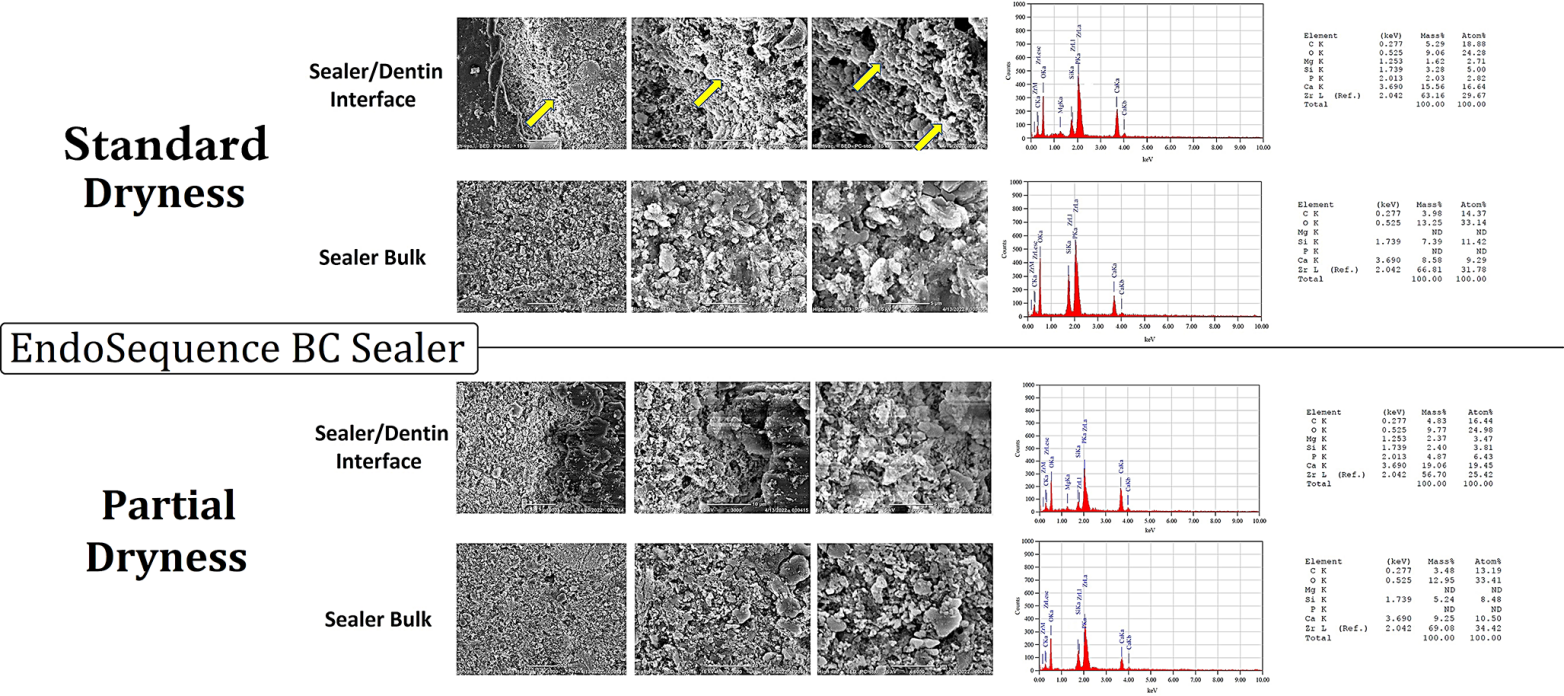
Composite figure showing SEM images (magnifications x1000, x3000 and x6000) of samples from the SC-ES-SD and SC-ES-PD groups (Standard dryness and partial dryness before obturation using EndoSequence BC as the sealer), as well as the EDX graph and the elemental composition, both at the interface and in the bulk of the sealer. The yellow arrows denote areas of more pronounced hydration that are more evident in proximity to the dentinal wall

**Fig. 6:**
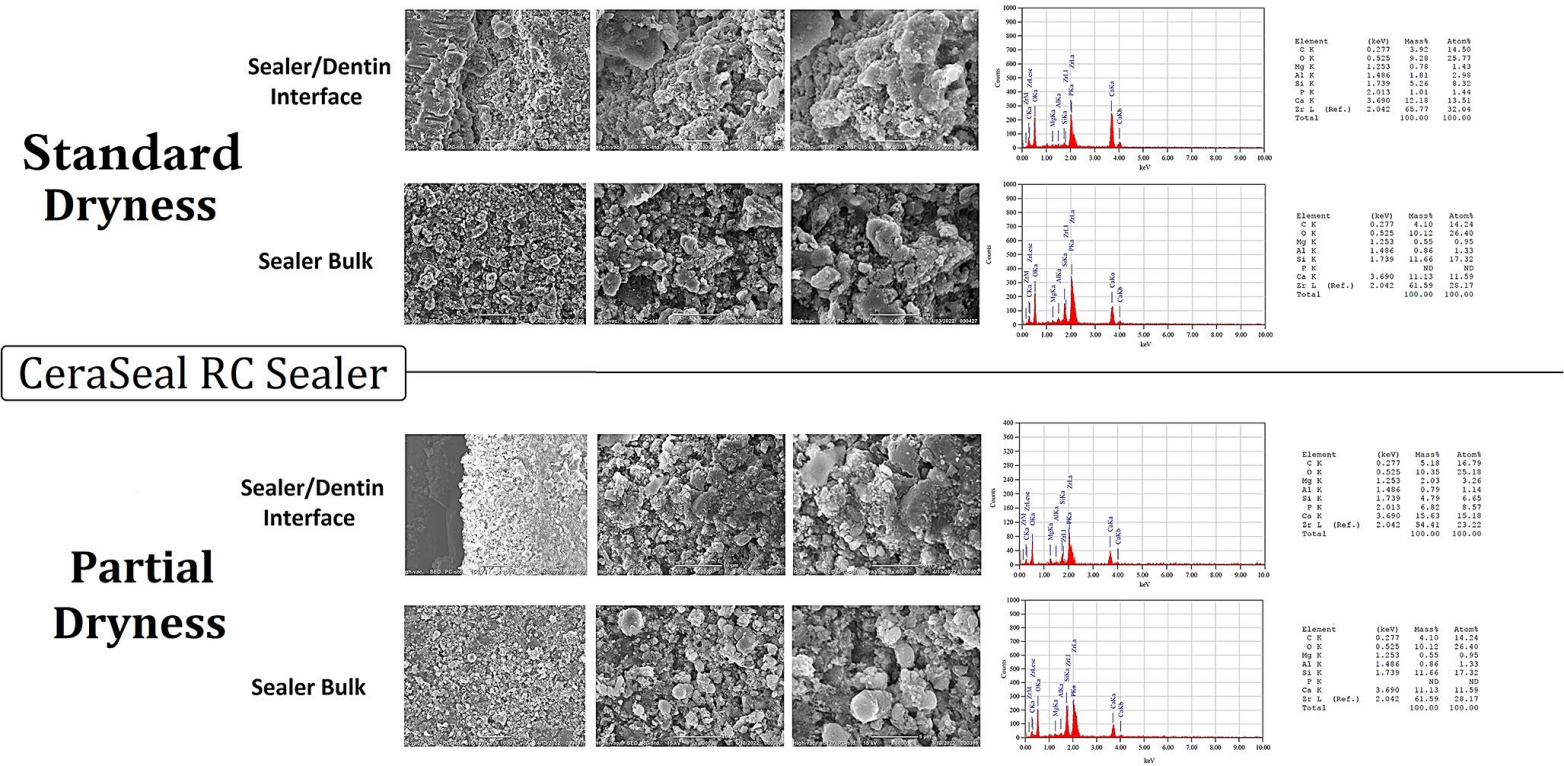
Composite figure showing SEM images (magnifications x1000, x3000 and x6000) of samples from the SC-CeS-SD and SC-CeS-PD groups (Standard dryness and partial dryness before obturation using CeraSeal as the sealer), as well as the EDX graph and the elemental composition, both at the interface and in the bulk of the sealer

Representative samples of the SEM images and EDX examination are given in Figs. 5 and 6. EDX examination of SC-ES-SD and SC-ES-PD (Figs. 5 and 6) revealed differences regarding the elemental composition between both sealers, as well as between the sealer layer at the dentinal interface and the bulk region away from the dentin for the same sealer. In both SC-ES-SD and SC-ES-PD samples, at the dentinal interface, Endosequence BC mainly showed zirconium (Zr), calcium (Ca), carbon (C), oxygen (O), magnesium (Mg), silica (Si) as well as phosphorous (P), whereas the bulk material showed no phosphorous (P) or magnesium (Mg). Also, the interface regions showed about double the calcium content compared to the bulk region. In both SC-CeS-SD and SC-CeS-PD samples (Figs. 5 and 6), CeraSeal contained aluminum (Al) and more magnesium (Mg) than Endosequence BC. The presence of phosphorous (P) at the interface and absence in the bulk noticed in SC-ES-SD and SC-ES-PD, was also notable with both SC-CeS-SD and SC-CeS-PD.

**Fig. 7:**
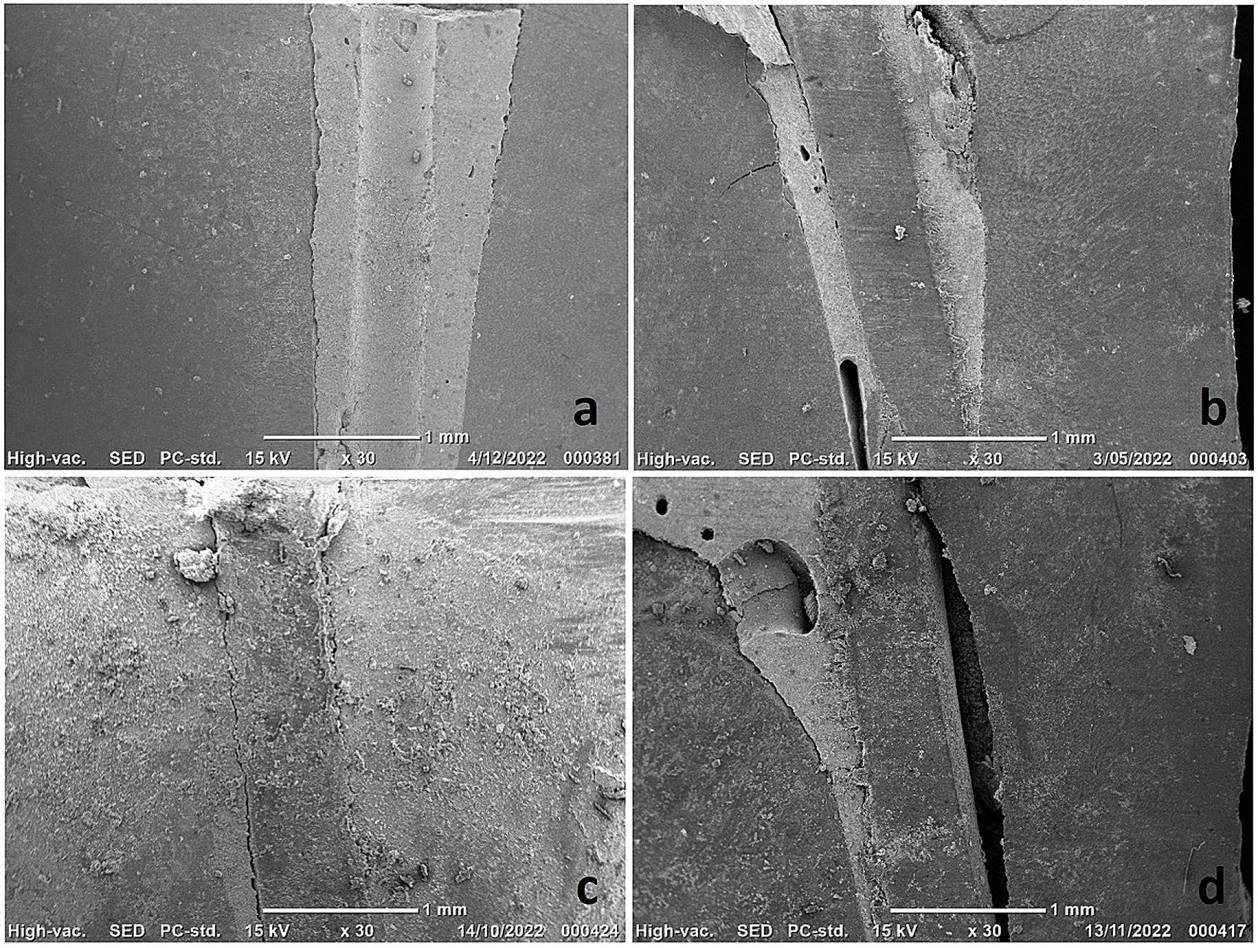
Composite figure showing sealer adaptation and interfacial gaps using SEM images (magnification X30): **(a)** a representative sample from the SC-ES-SD group, **(b)** a representative sample from the SC-ES-PD group, **(c)** a representative sample from the SC-CeS-SD group, and **(d)** a representative sample from the SC-CeS-PD group. (SC-ES-SD: Standard dryness before obturation using Endosequence BC as the sealer, SC-ES-PD: Partial dryness before obturation using Endosequence BC as the sealer, SC-CeS-SD: Standard dryness before obturation using CeraSeal as the sealer, SC-CeS-PD: Partial dryness before obturation using CeraSeal as the sealer)

Regarding SEM examination, crystalline particles were found on the surface of both sealers. SEM images of x30 magnification also showed that the sealer in SC-ES-SD samples showed less cracking than in the SC-CeS-SD samples and adapted slightly better to both interfaces; the gutta-percha-sealer interface and the sealer-dentinal interface (Fig. 7). Voids were evident at both interfaces with both sealers in both SC-ES-PD and SC-CeS-PD samples (Fig. 7).

## Discussion

An ideal root canal obturation that efficiently fills the root canal and entombs any lingering bacteria is the targeted treatment’s culmination that maintains long-term periradicular health [2]. Through decades, various classes of endodontic sealers were developed to try and provide the permanent seal that complements a successful cleaning and desinfection protocol [14]. However, clinical postoperative evaluation has a limited capacity to estimate efficient obturation and is merely dependent on the radiographic appearance of the obturation white lines [2]; a perception that was shattered when the beautiful “white lines” of Resilon had more than quintuple the odds of failure [5]. Regardless of how marketing still employs the radiographic density of a sealer as a mark of supremacy, the academic community is now aware of the importance of stable obturation to a successful root canal treatment.

HCSBS have many favorable properties that can make them represent the long-sought optimum endodontic sealers. Given their reportedly excellent flow [51], they have been linked frequently with the single-cone obturation technique. This approach that employs a matched gutta-percha point is simple, time-saving, and less operator-dependent [52]. However, such a compaction-free technique might not seal the root canal system properly, especially coronally, and its efficiency still lacks evidence. Also, the definition of the best hydration conditions before sealer application in clinical settings is yet unexplored. This is important because HCSBS need water for their setting reaction [7]. This is not only a difference but rather the opposite of the hydrophobic nature of previous classes of sealers especially the popular epoxy resin-based sealers [32]. Therefore, the first phase of this study was designed to study the effect of the aforementioned variables under clinical conditions.

The quality of root canal obturation in this study was assessed by 2D and 3D measurements. While numerous studies employed the overall analysis of the percentage of voids by comparing the volume of obturating materials to that of the prepared canal space [10, 49], it was postulated that this approach causes an inconsistency in outcomes [32]. Thus, both 2D and 3D assessments were performed to obtain more reliable outcomes.

The first control group of this study included teeth obturated with the lateral compaction technique and the epoxy resin-based sealer AH Plus. AH Plus has been widely recognized as the benchmark against which newly introduced sealers are appraised [11]. The lateral compaction technique was used in this study as it is the most widely taught and used one, and because warm techniques are not suitable for some hydraulic sealers [53, 54]. Conventional gutta-percha was used because it is more commonly used, readily available, and more importantly because a previous study showed that gutta-percha coated with BC nanoparticles exhibited more interfacial gaps with the sealer [31]. These choices were made targeting results-generalization and to facilitate comparisons to existing and future studies. Moreover, the cold lateral compaction group CLC-ES-SD served as a second control group to assess if, when standard canal dryness was ensured prior to obturation, the obturation technique itself had an impact on the percentage of voids.

The results of this study showed significantly less voids in the control group obturated with AH Plus when compared to the hydraulic sealer, thus the null hypothesis was rejected. This observation agrees with the results of previous studies [32, 49, 55] but is in contrast with other findings reporting less or similar amounts of voids with calcium silicate-based sealers after obturation [9, 12, 22, 31, 52, 56, 57]. This can be attributed to the variations in the studies conditions regarding the use of extracted teeth, different obturation techniques, different imaging techniques, different sealer brands, and different setting environments.

The obturation technique had no significant effect on the incidence of voids when using the HCSBS and when the root canal was dried thoroughly using paper points before obturation. These findings are in agreement with those of Eltair et al. [31], but differ from those of Zhang et al. [46]. who found more voids when using the single cone technique. This may be attributed to the fact that their study focused on the filling of artificial canal isthmi. However, both studies agree with the general finding in literature that no obturation technique could achieve a void-free homogenous root canal filling regardless of the type of sealer used.

As in the present study, no significant differences regarding the percentage of voids were obtained between the two obturation techniques (cold lateral compaction versus single-con) when standard canal dryness was ensured (Table 2) it can be concluded that moisture conditions affected the performance of the hydraulic sealer in a significantly negative manner when using the single-cone technique. Samples with partially dried canals showed significantly more voids than those with fully dried canals (Table 2). Previous studies employed bond strength testing as a surrogate outcome to assess the effect of moisture on the quality of root canal obturation. Frasquetti et al. [27] found that completely drying the root canals with paper points yielded higher bond strength when using two types of hydraulic sealers, while Razmi et al. [29] found that residual moisture did not affect the bond strength of EndoSequence BC. Another study that investigated the effect of moisture on the bond strength of different sealers [26] recommended leaving canals slightly moist before filling. These contradictory findings highlight the need for a standardized model to assess the setting kinematics of HCSBS clinically.

The significant negative effect of an intentionally moist root canal just before obturation mandated further examination of whether inherent dentinal moisture is enough to ensure the complete setting reaction of HCSBS, and also to examine the resulting byproducts, their distribution, and significance. The environmental interaction of these sealers has considerable importance as it results in changes in their surface chemistry, and may even cause failure to set, thus having detrimental effects on the material and its function [58]. This reaction is unique and even chemically comparable sealers respond differently to the presence of moisture and dentinal fluids [59]. Because of the nature of their setting reaction, it was postulated that partially drying the root canal before obturation can be advantageous when HCSBS are used [30, 60]. However, this theoretical assumption has not been supported by sufficient clinical evidence yet.

Regarding the sealers’ composition, it is worth mentioning that no attempts were made in the present study to investigate the composition of the sealers in-vitro, as was already done in several previous studies [20, 53, 59, 61, 62]. The present study focused on investigating the clinical performance of two sealers, with different levels of pre-obturation intraradicular moisture. There were differences between both sealers even though they are both calcium silicate-based sealers. The presence of aluminum in CeraSeal and its absence in Endosequence BC agrees with the manufacturers’ statements [59]. However, the presence of carbon in both sealers was not disclosed. This finding agrees with the results of previous studies, conducted under in-vitro conditions on Endosequence BC [59, 63].

Regardless of the pre-obturation dryness protocol and the type of sealer, both sealers exhibited evident microscopic and structural variations between the sealer/dentin interface and the bulk of the sealer or the sealer/gutta-percha interface. Thus, the second null hypothesis was rejected. At the sealer/dentin interface, both sealers showed evident hydration products, with layers containing phosphate overlying the structure and masking some other sealer elements like the zirconia radiopacifier. Identification of phosphorus at the dentinal interface only and along with the higher amount of calcium can be attributed to the continuous hydration from the dentinal fluid inside the root canal. The bioactivity of both sealers is evident by the formation of unidentified apatite-like compounds at the sealer/dentin interface. However, given the lack of phosphate buffer solution, it is not permissible to label these compounds as hydroxyapatite [64].

The percentage of phosphate at the sealer/dentin interface was higher in the samples with partial dryness, however, the authors would still recommend total dryness before obturation because residual moisture negatively affected sealers’ adaptation and resulted in the presence of voids both at the interface and within the bulk of the sealer as shown by SEM images (Fig. 4) and by the results of the first part of this study. In routine root canal treatments, reducing voids supersedes the bioactivity of the sealer because residual voids and inaccessible anatomy can shelter microbes and predispose to treatment failure [65], so while the formation of hydration products denotes the presence of a strong impregnable layer of true chemical reaction it does not ensure 360⁰ adaptation. Secondly, given that phosphate compounds were still found at the interface in the total dryness groups, it can be presumed that the hydration process, being mediated by dentinal fluids, proceeds as long as the tooth remains in situ, thus deeming it unjustifiable to jeopardize adaptation with partial moisture seeking higher levels of bioactivity, however, this needs to be confirmed by further studies.

Even though this study’s sole focus was the performance of HCSBS, the observation that the control group employing AH Plus demonstrated significantly fewer voids should not be ignored. This highlights that HCSBS still need a better delivery system so that clinicians can harness their advantages along with the superior adaptation possible with AH Plus.

Up to our knowledge, there are no data available about the sealer hydration reaction assessed after clinical performance as done in this study, where the sealers were allowed to set surrounded by the dynamic dentinal moisture and were subjected to the fluctuating stresses of functional occlusion and thermal variations, thus taking the testing conditions to another level beyond the limitations of in-vitro testing and artificial teeth. This justifies the contradiction of some data compared to other studies using different storage media and lacking normal dentinal fluid flow and its renewal. However, the present study also has limitations; the study had a limited sample size, and was limited to premolars. Whether or not the clinical performance of the sealer would be different in molars (generally narrower canals with more complexities and curvatures as well as a large number of lateral and accessory canals [48, 66] that can be the source of continuous hydration) requires further investigations. Also, the study did not assess the effect of thermal-based obturation techniques, because very few HCSBS can tolerate heat [7, 54, 67] and the authors would rather invest the available samples in more generalizable groups, given the very restricted recruitment process. Finally, all samples belonged to a very limited age group of the included sample; whether aged dentin with probably a reduced water content and smaller diameters of dentinal tubules would yield different results also needs further investigation [68, 69]. However, the recruitment process of patients dictated these limitations because of ethical considerations [2]. Thus, future studies are needed to draw comparisons that were not covered in this study, and multicenter replications of this extensively reported study are needed to pile sufficient samples for strong meta-analyses and conclusive results.

## Conclusions

Within the limitations of this study, when using HCSBS the single-cone technique using a matching gutta-percha-cone performed comparable to the CLC technique, and standard (total) canal dryness should be attempted before obturation to minimize the incidence of intracanal voids. Also, standard canal dryness did not hinder the hydration of HCSBS at the dentinal interface.

## Data Availability

No datasets were generated or analysed during the current study.
